# Anti-Neuroinflammatory Effects of the Calcium Channel Blocker Nicardipine on Microglial Cells: Implications for Neuroprotection

**DOI:** 10.1371/journal.pone.0091167

**Published:** 2014-03-12

**Authors:** Bor-Ren Huang, Pei-Chun Chang, Wei-Lan Yeh, Chih-Hao Lee, Cheng-Fang Tsai, Chingju Lin, Hsiao-Yun Lin, Yu-Shu Liu, Caren Yu-Ju Wu, Pei-Ying Ko, Shiang-Suo Huang, Horng-Chaung Hsu, Dah-Yuu Lu

**Affiliations:** 1 Graduate Institute of Clinical Medical Science, China Medical University, Taichung, Taiwan; 2 Neurosurgery Department, Taichung Tzu Chi Hospital, Buddhist Tzu Chi Medical Foundation, Taichung, Taiwan; 3 School of Medicine, Tzu Chi University, Hualien, Taiwan; 4 Department of Bioinformatics, Asia University, Taichung, Taiwan; 5 Department of Cell and Tissue Engineering, Changhua Christian Hospital, Changhua, Taiwan; 6 Department of Genetics and Complex Diseases, Harvard School of Public Health, Boston, United States of America; 7 Department of Biotechnology, Asia University, Taichung, Taiwan; 8 Department of Physiology, School of Medicine, China Medical University, Taichung, Taiwan; 9 Department of Life Sciences, National Chung Hsing University, Taichung, Taiwan; 10 Graduate Institute of Basic Medical Science, China Medical University, Taichung, Taiwan; 11 Department of Medical Laboratory Science and Biotechnology, China Medical University, Taichung, Taiwan; 12 Department of Pharmacology and Institute of Medicine, College of Medicine, Chung Shan Medical University, Taichung, Taiwan; 13 Department of Orthopedic Surgery, China Medical University Hospital, Taichung, Taiwan; 14 Graduate Institute of Neural and Cognitive Sciences, China Medical University, Taichung, Taiwan; Kaohsiung Chang Gung Memorial Hospital, Taiwan

## Abstract

**Background/Objective:**

Nicardipine is a calcium channel blocker that has been widely used to control blood pressure in severe hypertension following events such as ischemic stroke, traumatic brain injury, and intracerebral hemorrhage. However, accumulating evidence suggests that inflammatory processes in the central nervous system that are mediated by microglial activation play important roles in neurodegeneration, and the effect of nicardipine on microglial activation remains unresolved.

**Methodology/Principal Findings:**

In the present study, using murine BV-2 microglia, we demonstrated that nicardipine significantly inhibits microglia-related neuroinflammatory responses. Treatment with nicardipine inhibited microglial cell migration. Nicardipine also significantly inhibited LPS plus IFN-γ-induced release of nitric oxide (NO), and the expression of inducible nitric oxide synthase (iNOS) and cyclooxygenase-2 (COX-2). Furthermore, nicardipine also inhibited microglial activation by peptidoglycan, the major component of the Gram-positive bacterium cell wall. Notably, nicardipine also showed significant anti-neuroinflammatory effects on microglial activation in mice *in vivo*.

**Conclusion/Significance:**

The present study is the first to report a novel inhibitory role of nicardipine on neuroinflammation and provides a new candidate agent for the development of therapies for inflammation-related neurodegenerative diseases.

## Introduction

Microglia play pivotal roles in host defense and tissue repair processes in the central nervous system [1]. Neuroinflammation caused by microglial activation have both beneficial and detrimental consequences in the nervous system [2], [3]. During neuroinflammation, activated microglia leads to clearance of debris or invading pathogens, and release of neurotrophic factors that regulate the microenvironment [4]. When sensing ATP leak from an injury site, microglia transform to a more motile state and migrate to the site of damage [5], which causes neuroinflammation and subsequent neurodegeneration [6]. However, hyperactivation of microglia results in the production of a variety of proinflammatory mediators, which have been implicated in the pathogenesis of several neurodegenerative diseases, including Alzheimer's disease [7], Huntington's disease [8], Parkinson's disease [9], [10], stroke [11] and hypoxia insults [12]. Microglial activation involves changes in cell morphology and the subsequent expression of new proteins, such as inducible nitric oxide synthase (iNOS) and cyclooxygenase-2 (COX-2). These proinflammatory cytokines have been shown to cause neuronal damage [13]–[16]. Increasing evidence has revealed that iNOS produces a sustained level of NO [17], and induction of COX-2 expression [18] in activated microglia which further aggravate the neuropathological processes. Previously, iNOS and COX-2produces detrimental effects in glial cells were also identified in the ischemic brain http://www.ncbi.nlm.nih.gov/pmc/articles/PMC3498223/ pone.0049701-Yermakova1 [19]. Consistent with this, focal ischemia-induced brain infarcts and associated neurological impairments are alleviated in iNOS null mice [20]. The expressions of the inflammation-related enzymes iNOS and COX-2 have also been reported in the striatum of Parkinson disease patients [21]. The expressions of TNF-α and IL-6 have also been found to be elevated in the brains of Alzheimer's disease patients [22], [23]. Although inflammatory mediators are necessary for normal neuronal cell functions, the microglial response must be tightly regulated to avoid over-activation and neurotoxic consequences [24].

Nicardipine is a dihydropyridine type voltage-sensitive calcium channel antagonist used in the treatment of vascular disorders. The mechanism and clinical effects of nicardipine closely resemble that of the other dihydropyridines. However, nicardipine is more selective for cerebral and coronary blood vessels and is widely used to manage severe hypertension after acute brain injury, including ischemic stroke, traumatic brain injury, and subarachnoid hemorrhage [25]–[27]. In clinical practice, nicardipine has also been used for the treatment of acute hypertension in acute brain disease, with promising outcomes [26], [28]–[31]. Previous studies also reported neuroprotective effects of nicardipine on hypertension-induced brain damage [32]–[34]. Few animal and human studies have assessed the beneficial use of nicardipine in acute ischemic stroke [35]. Notably, nicardipine has also been reported to regulate calcium signaling in glial cells [36], [37]. Previous studies also reported that the L-type calcium channel blockers verapamil [38] and nimodipine [39] induce anti-inflammatory effects in microglia. However, the actual protective and anti-inflammatory mechanism of nicardipine is unclear. In the present study, we addressed whether, in addition to controlling blood pressure, nicardipine also regulates neuroinflammatory responses in microglia and exerts further neuroprotective effects.

## Material and Methods

### Ethics statement

The protocol used for the experimental mice was reviewed and approved by the Institutional Animal Care and Use Committee of the China Medical University (IACUC approval no. CMU-102-16N). All animal studies were conducted according to institutional guidelines (Affidavit of Approval of Animal Use Protocol, No. 102-16N) approved by the Institutional Animal Care and Use Committee (IACUC) of China Medical University (Taichung, Taiwan).

### Reagents and antibodies

Recombinant murine IFN-γ was purchased from PeproTech (Rocky Hill, NJ). LPS from *Escherichia coli* Serotype 055:B5 was obtained from Sigma-Aldrich (St. Louis, MO). Peptidoglycan from *Staphylococcus aureus* was purchased from Fluka (Buchs, Switzerland). The antibody against ionized calcium binding adaptor molecule 1 (Iba 1) was purchased from Wako Pure Chemical Industries (Osaka, Japan). Primary antibodies against β-actin, p65, ERK2, phosphorylated ERK1/2, p38, and JNK were purchased from Santa Cruz Biotechnology (Santa Cruz, CA). Primary antibodies against phosphorylated p38, phosphorylated JNK, and phosphorylated p65 were purchased from Cell Signaling and Neuroscience (Danvers, MA). The primary antibody against iNOS was purchased from BD Transduction Lab (Lexington, KY). The primary antibody against COX-2 was purchased from Cayman Chemicals (Ann Arbor, MI).

### Cell culture

The murine microglial cell BV-2 was originally generated by infecting primary microglial cell cultures with a v-raf/v-myc oncogene carrying a retrovirus (J2). BV-2 microglia retain the morphological, phenotypical, and functional properties of freshly isolated microglial cells (Blasi *et al*, 1990). Cells were cultured in DMEM (Gibco, Grand Island, NY) with 10% FBS at 37°C, and passaged by trypsinization.

### Animals

All mice were manipulated in accordance with the Animal Care and Use Guidelines of the China Medical University (Taichung, Taiwan). Male ICR mice were purchased from the National Laboratory Animal Center (Taipei, Taiwan). The mice were housed in a temperature- and humidity-controlled environment, and given free access to foods and water. All studies involving animals are reported in accordance with the ARRIVE guidelines (animals in research: reporting in vivo experiments) [40]. Mice were acclimated to their environment for at least 7 days before conducting the experiments.

### Protocols of treatment

Nicardipine was dissolved in DMSO at 10 mM as stock solution. The concentrations of nicardipine (1–10 μM) used in cell culture of this study were according to previous report [41]. Cells were treated with nicardipine or vehicle control for 60 min and then stimulated with ATP, LPS/IFN-γ or peptidoglycan. Cell migration assay was determined after ATP treatment for 24 h. Inflammatory mediators were determined by real-time PCR after LPS plus IFN-γ treatment for 6 h. Nitric oxide, iNOS and COX-2 expressions were measured after LPS/IFN-γ or peptidoglycan treatment for 24 h. Phosphorylated protein levels were determined after LPS/IFN-γ treatment for 60 min. In the immunohistochemical and flow cytometry experiments, mice were administered saline or nicardipine by intraperitoneal injection for 3 consecutive days. Concentrations of nicardipine administrated to mice were according to previous study [42]. On the third day, 2 h after the injection of saline or nicardipine, mice were injected with LPS intraperitoneally and housed for 24 h before euthanized.

### Tissue preparation and immunohistochemistry

Eight-week-old mice received an intraperitoneal injection of saline or LPS (20 mg/kg) (*E. coli*, serotype 0127:B8). Twenty-four hours later, mice were deeply anesthetized with chloral hydrate and transcardially perfused with 10% formaldehyde. The brains were dissected and post-fixed overnight, then incubated in a 30% sucrose solution at 4°C until they sank. Coronal serial sections (30 μm) were sliced on a freezing sliding microtome cryostat (Leica CM305S). Endogenous peroxidases in the free-floating slices were quenched using hydrogen peroxide. Brain sections were blocked by nonspecific binding with goat serum, permeabilized with Triton X-100, and then incubated with primary antibodies against Iba-1, overnight at 4°C. Following incubation with a biotinylated secondary antibody, the sections were incubated with an avidin-biotin complex (Vector Laboratories), and labeling was visualized with diaminobenzidine (DAB). The cerebral cortex, hippocampus and striatum were digitally captured at 200× magnifications using a light microscope.

### Flow cytometry

Microglial isolation from mouse brain [43] and flow cytometry analysis [44], [45] were performed according to our previous reports. Briefly, mice were perfused with a phosphate buffered solution (PBS). Brains were homogenized in a digestion buffer, then homogenates were separated by a discontinuous Percoll (GE-healthcare, Uppsala, Sweden) density gradient (70%, 37%, 30%, and 0%). The gradient was centrifuged for 40 min at 200 × g and microglia were collected from the interphase between the 70% and 37% Percoll layers.

The surface antigens of microglial cells were assayed by flow cytometry. Fc receptors were blocked with anti-CD16/CD32 antibody (eBioscience, San Diego, CA) for 30 min at 37°C. Microglia were incubated with anti-CD11b conjugated to FITC and anti-CD45 conjugated to peridinin-chlorophyll protein-cyanine5.5 (PerCP-Cy5.5) antibodies (BD Biosciences, San Jose, CA). Expression of these surface receptors was determined using a Becton-Dickinson FACSCaliburTM four-color cytometer (Becton Dickinson, San Jose, CA). Ten thousand events were recorded and microglia were identified by CD11b^+^ and CD45^low^ expression. Previous report demonstrated that these relative percentages of cells do not change with LPS [46].

Intracellular IL-6 and TNF-α production by microglia were determined by intracellular flow cytometric analysis based on a BD Cytofix/Cytoperm™ Plus fixation/permeabilization protocol (BD Biosciences, San Jose, CA). After blocking, cells were stained with anti-CD11b-FITC and anti-CD45-PerCP-Cy5.5 antibodies. Next, cells were fixed and permeabilized with BD Cytofix/Cytoperm™ solution for 20 min. Cells were washed with BD Perm/Wash™ buffer, re-suspended in BD Perm/Wash™ buffer, and incubated with either anti-IL-6-PE and anti-TNF-α-APC (BD Biosciences, San Jose, CA) for 30 min. Cells were then washed twice in BD Perm/Wash™ buffer and re-suspended in FACS buffer.

### Western blot analysis

Western blot was performed according to our previous report [47]. Briefly, cells were lysed in a homogenization buffer for 30 min on ice, and equal amounts of the samples were loaded in each lane. The membranes were blocked with non-fat milk and then probed with primary antibodies. After undergoing several washes, the membranes were incubated with peroxidase-conjugated secondary antibodies. The blots were visualized by enhanced chemiluminescence using Fuji medical X-ray film (Fujifilm, Tokyo, Japan). The blots were then stripped by incubation in stripping buffer and reprobed a loading control. Quantitative data were obtained using a densitometer and Image J software (National Institute of Health, Bethesda, MA).

### Migration assay

*In vitro* migration assay was performed using Costar Transwell inserts (Pore size: 8 μm; Corning, Albany, NY) in 24-well plates as described previously [48]–[50]. Approximately 1 × 10^4^ cells in 200 μl of serum-free medium were placed in the upper chamber, and 300 μl of the same medium containing ATP was placed in the lower chamber. Before performing the migration assay, cells were pre-treated for 60 min with nicardipine followed by treatment with ATP during the 24-h migration assay (incubated at 37°C in 5% CO_2_). After the 24-h assay, the cells were stained with 0.05% crystal violet and 2% methanol. Non-migratory cells on the upper surface of the filters were removed by wiping with a cotton swab. Cell number was counted in five random fields per well under a microscope at 200× magnification. Images of migratory cells were observed and acquired using a digital camera and light microscope.

### Quantitative real-time PCR

Quantitative real-time PCR was performed according to our previous report [51]. Briefly, quantitative real-time PCR using SYBR Green Master Mix was performed with StepOne Plus System (Applied Biosystems, Singapore). After incubation at 50°C for 2 min and 95°C for 10 min, the PCR was performed as follows: 40 cycles at 95°C for 10 s and 60°C for 1 min. The threshold was set above the non-template control background and within the linear phase of target gene amplification to calculate the cycle number at which the transcript was detected (denoted as CT).

### Nitric oxide assay

Production of nitric oxide was assayed by measuring nitrite levels, the stable product of nitric oxide, in the culture medium, as described in our previous report [52]. Briefly, the accumulated nitrite in the medium was determined by a colorimetric assay with a Griess reaction. The culture supernatant reacted with an equal volume of Griess reagent (0.1% naphthylethylenediamine and 1% sulfanilamide in 5% H_3_PO_4_). After 10 min at room temperature in the dark, the absorbance was determined at 550 nm using a microplate reader (Thermo Scientific, Vantaa, Finland).

### MTT assay

Cell viability was determined using the 3-(4,5-dimethylthiazol-2-yl)-2,5- diphenyltetrazolium bromide (MTT) assay [53]. After treatment with nicardipine for 24 h, cell culture media were removed and washed with PBS. MTT (0.5 mg/ml) was added to each culture well and the mixture was incubated for 2 h at 37 °C. The MTT reagent was then replaced with DMSO (100 μl per well) to dissolve formazan crystals. After the mixture was shaken at room temperature for 10 min, absorbance was determined at 550 nm using a microplate reader (Thermo Scientific, Vantaa, Finland).

### Statistical analyses

The values are reported as mean ± S.E.M. Statistical analyses for two groups were performed using Student's *t-*test. Statistical comparisons of more than two groups were performed using one-way ANOVA with Bonferroni's *post-hoc* test. The difference was determined to be significant if the *p* value was <0.05.

## Results

### Nicardipine suppresses neuroinflammatory responses in microglial cells

**Figure 1:**
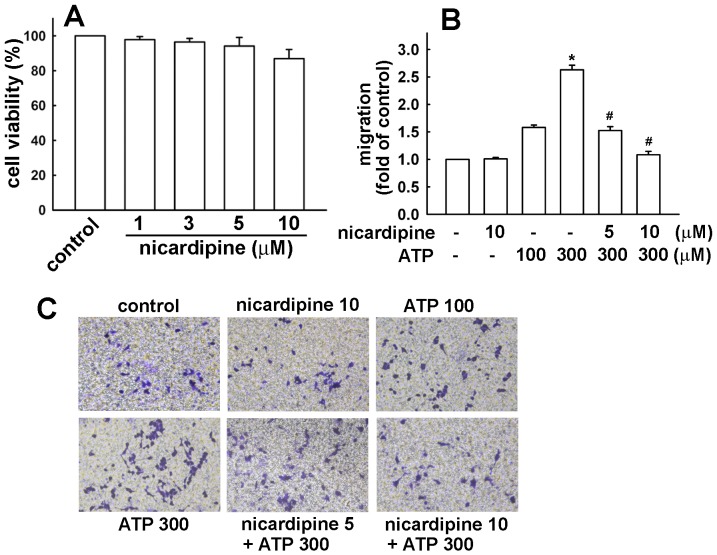
Effect of nicardipine on ATP-induced microglial cell migration. (A) Cell viability following nicardipine treatment in BV-2 microglia. Cells were treated with concentrations ranging from 1 to 10 μM of nicardipine for 24 h, and cell viability was measured by the MTT assay. The results are expressed as mean ± S.E.M. of three independent experiments. (B) Cells were pre-incubated with or without nicardipine (10 μM) for 60 min followed by a 24-h treatment with ATP (100 or 300 μM). *In vitro* migratory activities were examined using a cell culture insert system. The results are expressed as mean ± S.E.M. from 4 to 5 independent experiments. *, *p*<0.05 compared with the control group; #, *p*<0.05 compared with the ATP (300 μM) alone. (C) The migrated cells were visualized by phase-contrast imaging.

**Figure 2:**
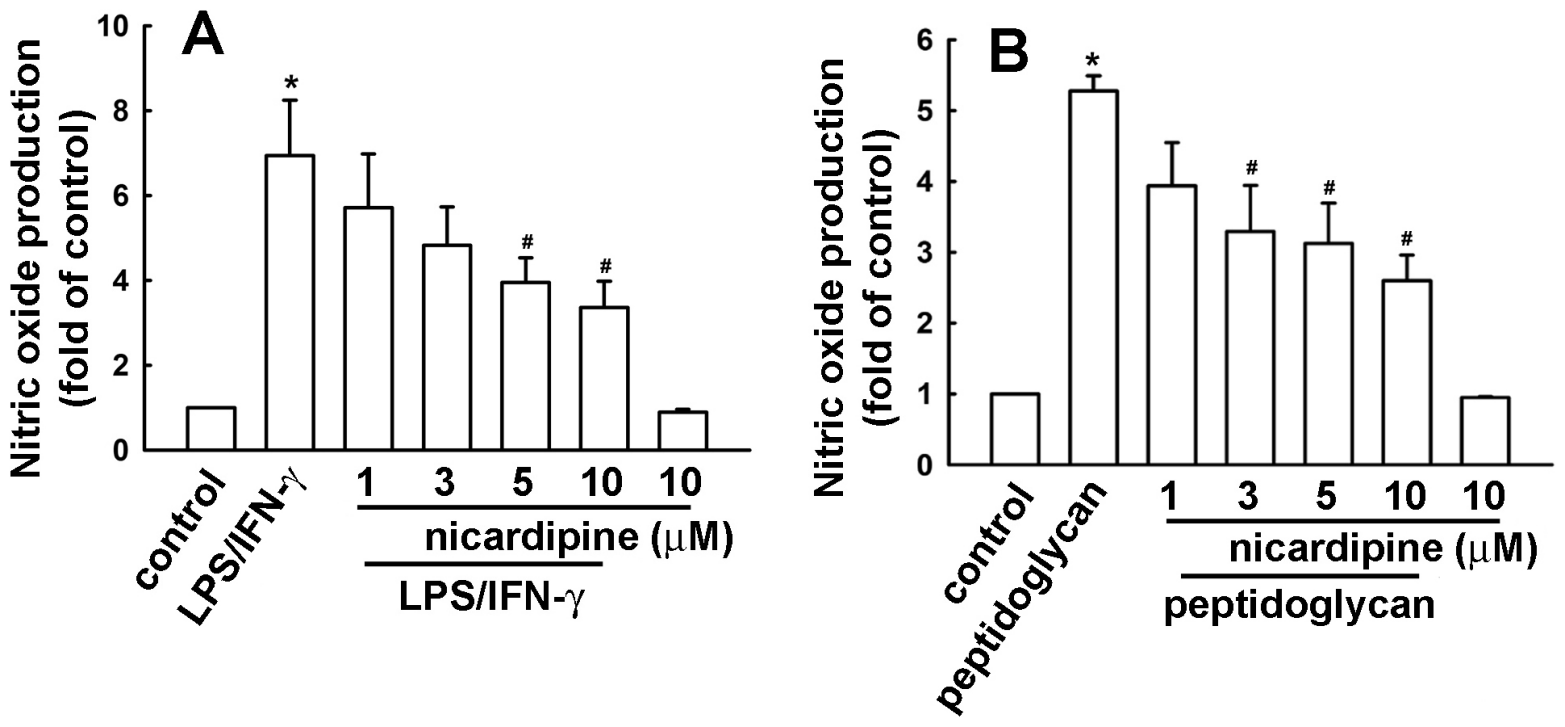
Inhibitory effect of nicardipine on LPS/IFN-γ or peptidoglycan-stimulated nitric oxide production. BV-2 microglial cells were pretreated with different concentrations of nicardipine (1, 3, 5, or 10 μM) for 60 min before application of LPS (10 ng/ml) plus IFN-γ (10 ng/ml) (A) or peptidoglycan (10 μg/ml; B) for another 24 h. The culture media were collected and analyzed by a Griess reaction. Nitric oxide production is significantly different between the LPS/IFN-γ (or peptidoglycan) treatment alone and the LPS/IFN-γ (or peptidoglycan) treatment with nicardipine groups (one-way ANOVA followed by Bonferroni's *post hoc* test). The results are expressed as mean ± S.E.M. from 3 to 4 independent experiments. *, *p*<0.05 compared with the control group; #, *p*<0.05 compared with the LPS/IFN-γ or peptidoglycan treatment.

**Figure 3:**
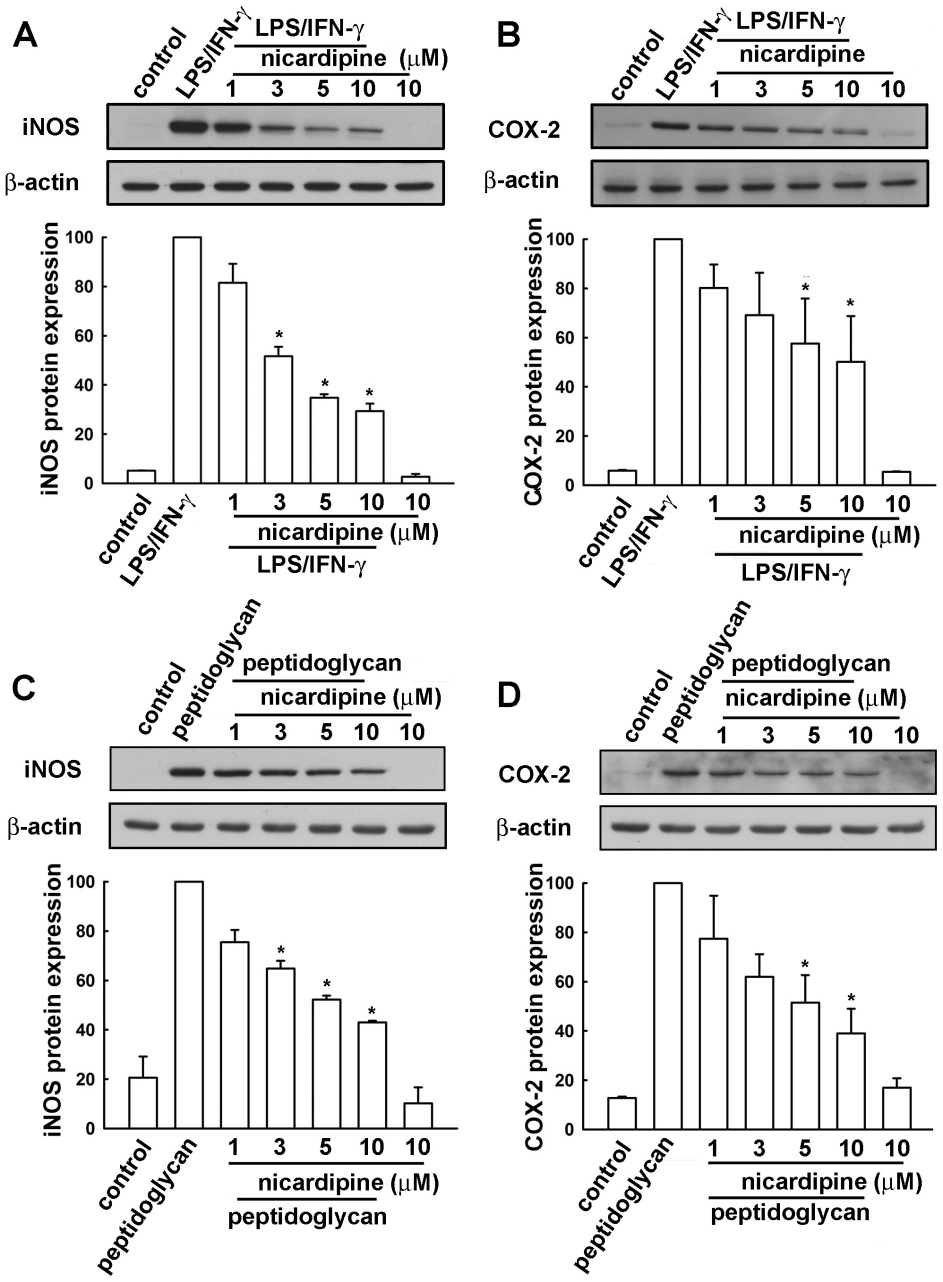
Inhibitory effect of nicardipine on LPS/IFN-γ- or peptidoglycan-stimulated iNOS and COX-2 expressions. (A and B) BV-2 microglial cells were pretreated with different concentrations of nicardipine (1, 3, 5, or 10 μM) for 60 min before application of LPS (10 ng/ml) plus IFN-γ (10 ng/ml) for another 24 h. (C and D) Cells were pretreated with different concentrations of nicardipine (1, 3, 5, or 10 μM) for 60 min before application of peptidoglycan (10 μg/ml) for another 24 h. Western blot analysis for iNOS (A and C) and COX-2 (B and D) expression was performed on whole cell lysates. The quantitative results are shown in the bottom panels. iNOS expression was significantly different between the LPS/IFN-γ (or peptidoglycan) treated-group and the group treated LPS/IFN-γ (or peptidoglycan) with nicardipine (one-way ANOVA followed by Bonferroni's post hoc test). COX-2 expression was significantly different between the LPS/IFN-γ (or peptidoglycan) treated- group and the LPS/IFN-γ (or peptidoglycan) with nicardipine treated- group (one-way ANOVA followed by Bonferroni's *post hoc* test). The results are expressed as mean ± S.E.M. from 4 to 5 independent experiments. *, *p*<0.05 compared with the LPS/IFN-γ or peptidoglycan treatment.

**Figure 4:**
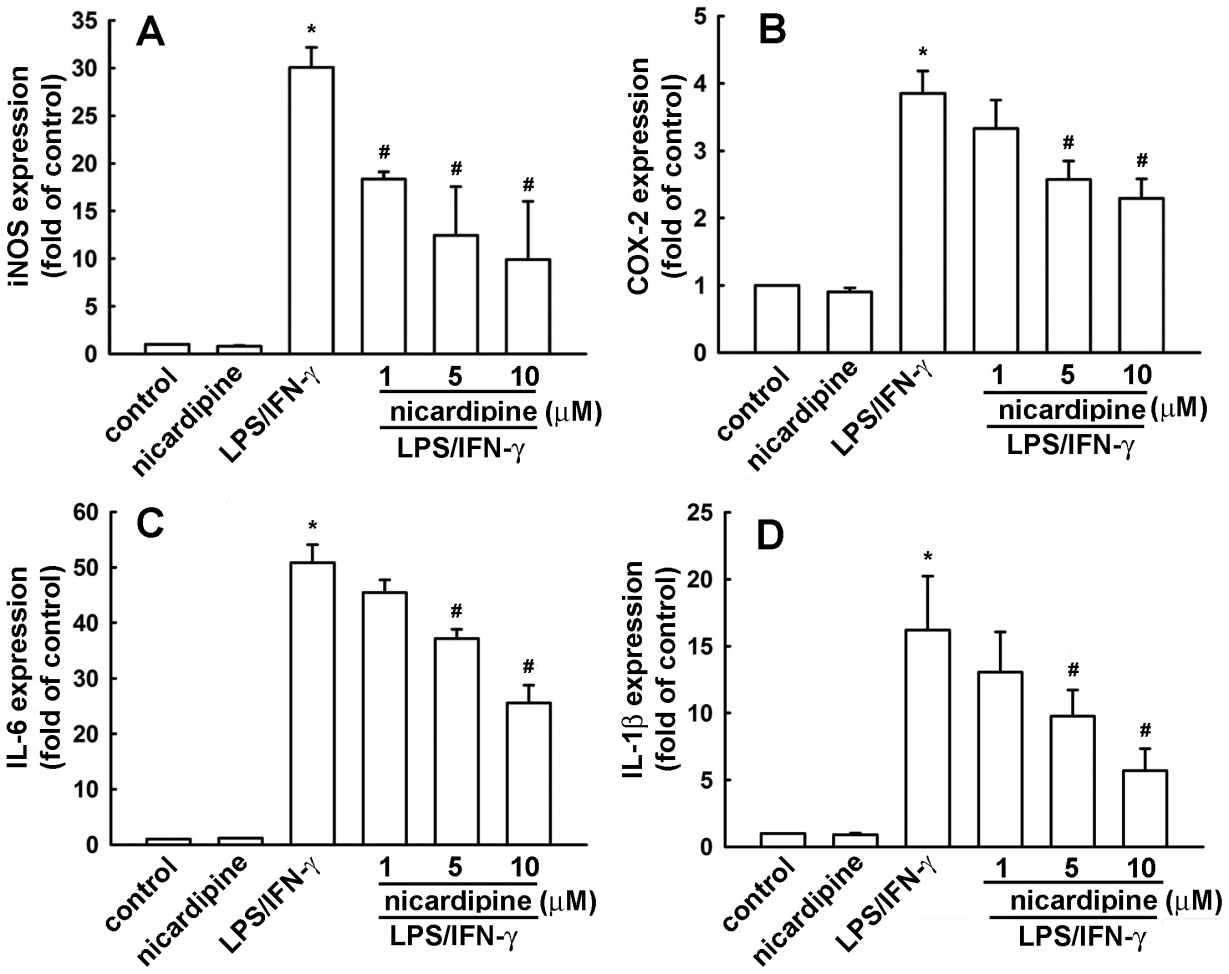
Nicardipine suppresses the expression of inflammatory mediators in BV-2 microglial cells. Cells were pretreated with different concentrations of nicardipine (1, 5, or 10 μM) for 60 min, then challenged with LPS (10 ng/ml) plus IFN-γ (10 ng/ml) for another 6 h. The expression of iNOS, COX-2, IL-6, and IL-1β (A, B, C and D respectively) were determined by real-time PCR. Cytokine expression was significantly different between the LPS/IFN-γ alone and the LPS/IFN-γ with nicardipine groups (one-way ANOVA followed by Bonferroni's *post hoc* test). The results are expressed as mean ± S.E.M. from 3 to 4 independent experiments. *, *p*<0.05 compared with the control group; #, *p*<0.05 compared with the LPS/IFN-γ treatment alone group.

We used BV-2 microglia to study the effects of nicardipine on neuroinflammatory responses. Concentrations ranging from 1 to 10 μM nicardipine were used. A colorimetric cell viability assay (MTT) confirmed that these concentrations did not affect cell viability (Fig. 1A). Nicardipine inhibited an ATP-induced increase in BV-2 microglial migratory activity (Fig. 1B). Representative micrographs of migrating cells are shown in Fig. 1C. Notably, nicardipine treatment alone did not affect baseline cell migratory activity. To determine the effect of nicardipine on nitric oxide production, cells were treated with different concentrations of nicardipine (1 to 10 μM) and were stimulated with LPS plus IFN-γ. The cell culture medium was then collected to determine the nitrite content. We have previously demonstrated that peptidoglycan, a component of the Gram-positive bacterium cell wall, causes neuroinflammation in microglia [54], [55]. Hence, to further determine the effect of nicardipine on nitric oxide production, BV-2 microglia were also stimulated with peptidoglycan. As shown in Fig. 2A and 2B, nicardipine effectively inhibited nitric oxide production in a concentration-dependent manner following exposure to either LPS (10ng/ml) plus IFN-γ (10ng/ml) or peptidoglycan (10 μg/ml). In addition, the inhibition of nitric oxide production initiated at 3∼5 μM achieved a maximum at 10 μM. The levels of iNOS expression were detected by western blotting. Nicardipine also reduced LPS/IFN-γ- and peptidoglycan-induced iNOS expression (Fig. 3A and 3C, respectively) and COX-2 expression in a concentration-dependent manners (Fig. 3B and 3D). Notably, nicardipine treatment alone did not affect iNOS, COX-2 and nitric oxide expression. We further analyzed the expression of inflammatory mediators by real-time PCR. BV-2 microglia were treated with different concentrations of nicardipine (1 to 10 μM) and stimulated with LPS plus IFN-γ for 6 h. Nicardipine potentiates a concentration-dependent suppression of iNOS and COX-2 when stimulating BV-2 with LPS/IFN-γ (Fig. 4A and 4B). Similarly, treatment with nicardipine also inhibited LPS/IFN-γ-induced IL-6 and IL-1β expression in a concentration-dependent manner (Fig. 4C and 4D, respectively). These results indicate that nicardipine exerts anti-inflammatory effects on microglial cells.

### Regulatory effects of nicardipine on signaling pathways

**Figure 5:**
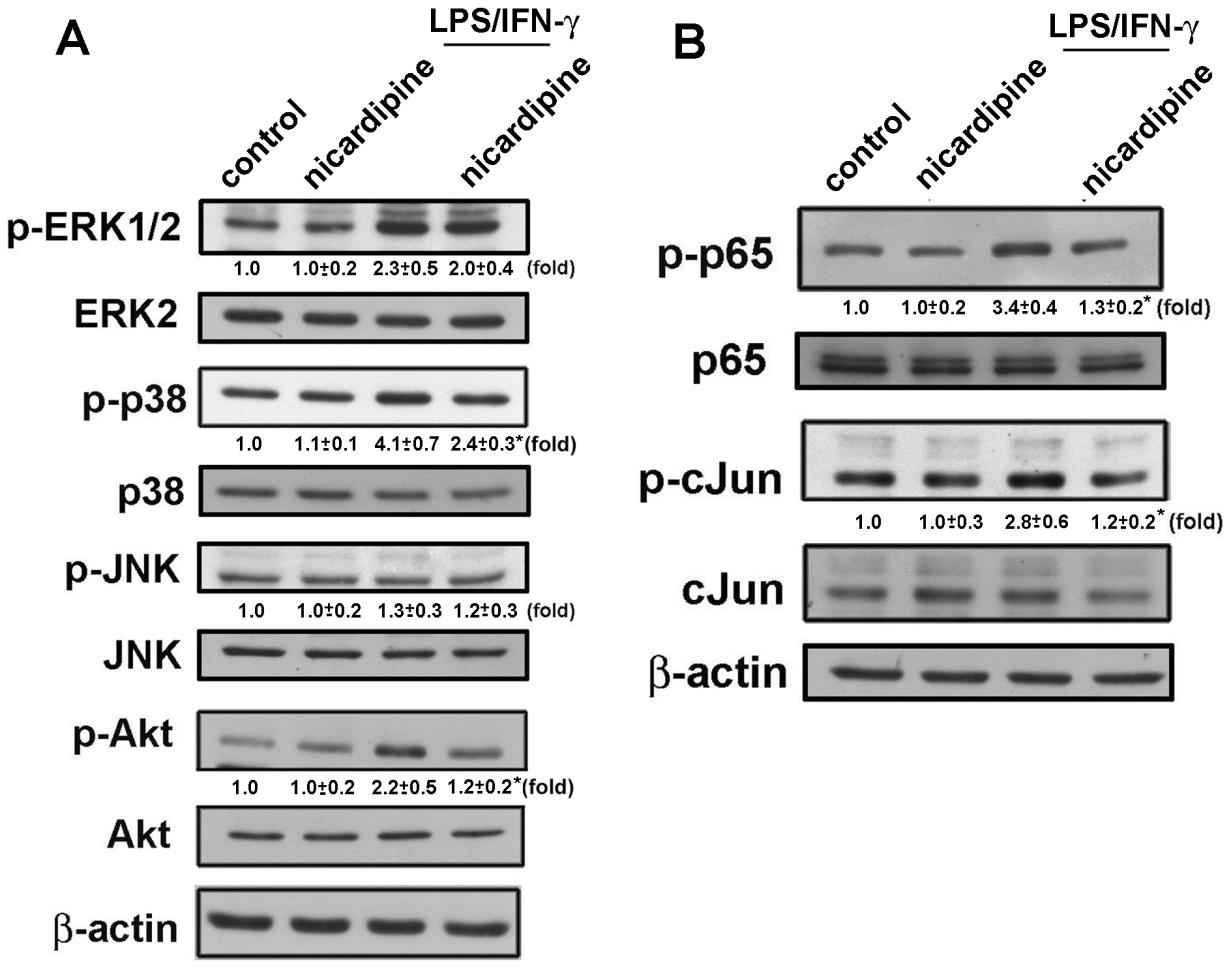
Nicardipine suppresses LPS/IFN-γ-induced signaling pathways. BV-2 microglial cells were pretreated with nicardipine (10 μM) for 60 min, then exposed to LPS (10 ng/ml) plus IFN-γ (10 ng/ml) for another 60 min. Western blot analysis was performed on whole cell lysates, and the signal intensities were normalized to total protein expression. The results are expressed as mean ± S.E.M. from 3 to 4 independent experiments. *, *p*<0.05 compared with the LPS/IFN-γ treatment alone group.

We further studied the signaling pathways involved in the inhibitory effects of nicardipine on neuroinflammation in microglia. MAP kinase and PI3 kinase/Akt are the most important signaling pathways in the regulation of inflammatory responses in macrophages and microglia [56]–[58]. We examined whether MAP kinase activation is modulated by nicardipine in microglia. BV-2 microglia were treated with nicardipine and then stimulated with LPS plus IFN-γ for 60 min. Western blot analysis for phospho-ERK, phospho-p38, phospho-JNK, or phospho-Akt was performed on whole cell lysates. As shown in Fig. 5A, nicardipine inhibited LPS/IFN-γ-induced p38 and Akt expression but not ERK and JNK. Additionally, nicardipine at 10 μM did not affect LPS/IFN-γ-induced MAP kinase and Akt phosphorylation. Expressions of inflammatory mediators are mainly regulated by the transcription factors NF-κB and AP-1 in microglia [52], [59]. As shown in Fig. 5B, LPS plus IFN-γ treatment increased p65 and cJun activation in BV-2 microglia after 90 min. The increases in p65 and cJun activation following LPS/IFN-γ treatment were attenuated by nicardipine treatment.

### Nicardipine inhibits proinflammatory cytokine expression in mouse brain microglia

**Figure 6:**
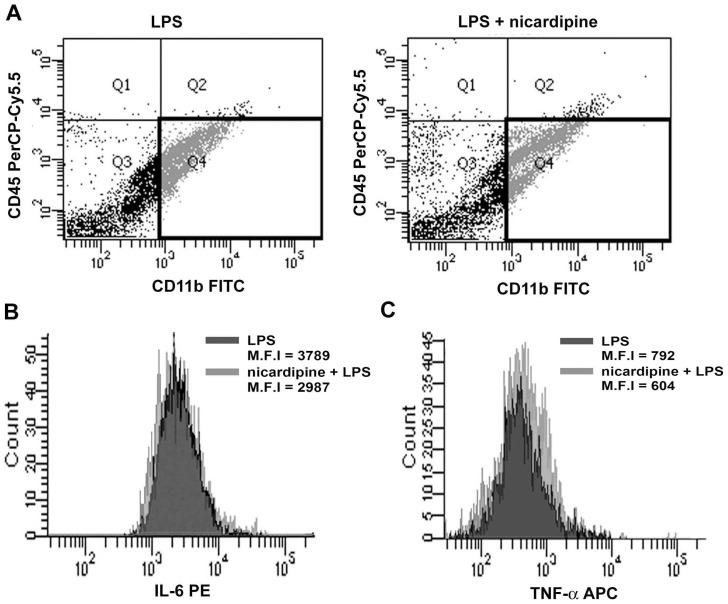
LPS-induced intracellular IL-6 and TNF-α expression on microglia is reduced by nicardipine. Mice received intraperitoneal injections of saline or nicardipine (5 mg/kg) for 3 consecutive days. On the third day, 2 h after injection of saline or nicardipine, mice were injected with LPS (5 mg/kg) and housed for another 24 h. (A) Representative bivariate dot plots of Percoll-isolated microglial cells stained with anti-CD11b-FITC and anti-CD45-PerCP-Cy5.5. Microglia were identified by CD11b^+^/CD45^low^ staining. Representative histograms of intracellular IL-6 (B) and TNF-α (C) expression in isolated microglia. Mean fluorescence intensity (M.F.I.) of intracellular IL-6 and TNF-α expressed by CD11b^+^/CD45^low^ microglia following experimental treatments.

We next examined whether nicardipine suppresses markers of microglial activation *in vivo*. Mice first received intraperitoneal injections of saline or nicardipine for 3 consecutive days, followed by an intraperitoneal injection of LPS 2 h after injected with saline or nicadipine on the third day. Twenty-four hours later, expression of surface CD11b/CD45 and intracellular IL-6 and TNF-*α* were determined by flow cytometry. As shown in Fig. 6A, the representative bivariate dot plots of Percoll-isolated microglia stained with CD11b^+^ and CD45^low^ confirmed the presence of this subset of microglia in mice receiving either LPS or co-treatment of nicardipine and LPS. The mean fluorescence intensity (M.F.I) reflecting intracellular IL-6 and TNF-*α* expression by microglia were reduced in the brains of mice that received LPS with nicardipine treatment (Fig. 6B and 6C).

### Nicardipine inhibits microglial activation in a mouse model

**Figure 7:**
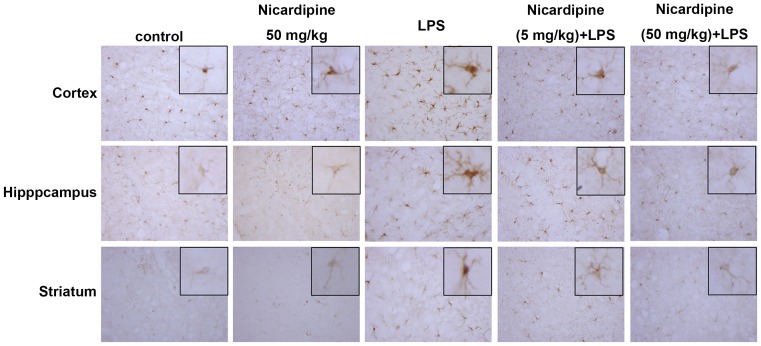
Nicardipine prevents LPS-induced microglial activation. ***Mice received*** intraperitoneal injections of nicardipine at concentrations of either 5/kg or 50 mg/kg, ***once per day***, for ***3 consecutive days. On the third day***, nicardipine treatment was followed with a single ***intraperitoneal injection*** of LPS (20 mg/kg). Microglial morphology was visualized by anti-Iba-1 immunolabeling and DAB (n = 5 each group).

To determine the improvements induced by nicardipine treatment on neuroinflammatory responses *in vivo*, we performed an immunohistochemical analysis on microglia. The clinical dose of nicardipine administration used for managing blood pressure is approximately 5 mg/kg [60]. Mice were continuously administered nicardipine at a dose of 5 or 50 mg/kg daily for 3 days, and were then injected with or without LPS. The activation of microglia was assessed morphologically by immunohistochemistry with the well-characterized Iba-1-specific antibody. Twenty-four hours after LPS injection, microglial activation was observed to be homogeneously distributed throughout the cortical, hippocampal and striatal regions. The Iba-1 immunolabeling was more intense in LPS-treated BV2 depicted with enlarger cell bodies and retracted processes compared with the control group (Fig. 7). Furthermore, LPS injection induced pronounced hypertrophy of cortical, hippocampal and striatal microglial cells. Microglial activation in the mouse brain was effectively attenuated by administration of nicardipine (Fig. 7). In addition, administration of nicardipine (50 mg/kg) alone did not affect microglia activation.

## Discussion

Nicardipine, a second-generation dihydropyridine-type calcium channel blocker, is the most frequently used anti-hypertensive agent following acute brain injury [26], [28], [31], [61]-[63]. Nifedipine and nimodipine are also established dihydropyridine-type calcium channel blockers. The protective effects of nimodipine have been evidenced by animal models of ischemic brain [64]. Previous studies have also reported that nicardipine can protect against hypertension-related brain damage [32]–[34]. Nifedipine has been reported to inhibit the expression of inflammatory and fibrogenic responses in advanced glycation end product-exposed fibroblasts [65]. The existence of calcium channels in glial cells is still controversial [66], [67]. Interestingly, the L-type calcium channel blockers nimodipine and verapamil, which have been reported to confer neuroprotective effects, and inhibit microglial activation [68]. Indeed, recent studies have reported that nimodipine and verapamil exert their neuroprotective effects through anti-neuroinflammatory properties [38], [69]. The anti-inflammatory effect of nimodipine has also been indicated to down-regulate TNF-α and IL-1β expression in the hippocampus [70] and IL-1β expression in microglia [39]. Importantly, the clinical dose of nicardipine used for managing blood pressure is approximately 5 mg/kg [60]. Here, we also found that 5 mg/kg of nicardipine inhibits microglia activation in our *in vivo* mouse model, but 50 mg/kg did not produce a more potent inhibitory response.

Cytokines are important mediators involved in immune, inflammatory, and immunomodulatory functions [71]. Although inflammatory responses are necessary for normal neuronal cell functions, microglial activation must be tightly regulated to avoid exaggerated responses and extended nenurotoxicity [24], [72]. Bacterial meningitis is the most frequently fatal infection in CNS, which results in significant neurological sequelae [73], [74]. In Gram-negative infections, LPS is a well-known activator of microglia. Peptidoglycan and lipoteichoic acid, the major components of the Gram-positive bacterium cell wall, activate microglia and induce the release of chemokines and cytokines [52], [54], [55]. Activated microglia migrate to the injury site and express inflammatory mediators, such as iNOS and COX-2, and these proinflammatory cytokines have been shown to result in neuronal damage [13]–[16]. In the present study, nicardipine effectively reduced cell migration of microglia induced by ATP, and proinflammatory cytokine expression activated by both LPS plus IFN-γ and peptidoglycan. Calcium channel blockers have been reported to act through a calcium channel-independent mechanism on multiple signaling molecules, including nuclear factor-kappa B [70], [75] and STAT3 [68]. Our present results and previous reports suggest that the neuroprotective effects of the calcium channel blocker nicardipine are mediated by a calcium channel-independent anti-neuroinflammatory effect.

Numerous lines of evidence support that MAP kinase and PI3 kinase/Akt are the most important signaling pathways to regulate inflammatory responses in microglia [52], [56], [58], [76]. Pro-inflammatory cytokines (e.g., interferon or interleukin) could activate microglial cells and trigger several inflammatory signaling pathways, including AP-1, Akt, NF-κB and MAP kinase. Here, we also report that nicardipine reduced the activation of the proinflammatory transcription factors NF-κB and AP-1. Nicardipine also inhibited LPS/IFN-γ-induced p38 and Akt activation in microglial cells. Our results also showed that nicardipine can inhibit microglial activation and intracellular expression of IL-6 and TNF-α *in vivo*. The results of current study suggest that nicardipine can exert anti-neuroinflammatory responses in microglia, which is unlikely to be mediated by blockade of calcium channels. The roles of calcium channels on microglia have led to considerable debate as to whether calcium buffering participates in neuroinflammatory responses, as calcium has been conceived to be physically relevant. However, the current findings offer new insights for developing therapeutic approaches to treat neuronal cell death and neuroinflammation-related disorders.
